# Developing a Tool for Monitoring and Evaluating a Network Approach to Innovation: Lessons from Year 1 of the SexEdVA Disability-Inclusive Sexual Health Network (DSHN)

**DOI:** 10.1007/s11121-023-01516-2

**Published:** 2023-04-21

**Authors:** Kim Hartzler-Weakley, Emma Duer, Kayla McKean

**Affiliations:** 1 SexEdVA, Institute for Innovation in Health and Human Services, James Madison University, Harrisonburg, VA USA; 2 Dainis & Company, Inc., Broadway, VA USA

**Keywords:** Adolescent sexual health, Disability, Evaluation, Innovation, Systems thinking, Program development

## Abstract

Disparities in sexual health outcomes for youth with disabilities require new and creative approaches to address the complex and often individualized barriers facing this diverse population. The Disability-inclusive Sexual Health Network (DSHN) establishes, funds, coordinates, and supports a multidisciplinary network of partners to explore, develop, test, refine, and evaluate innovative interventions that will improve optimal health, prevent teen pregnancy, and address sexually transmitted infections (STIs) for youth with disabilities in Virginia. DSHN developed the Monthly Reporting Instrument (MRI) to support communication, coordination, monitoring, and evaluation of the project, and shares findings from data collected using this tool. A mixed method analysis was conducted on data collected in year 1 using the MRI. A total of 67 MRI submissions were collected across eight DSHN Partners between March 2021 and March 2022. Analysis of the year 1 MRI data gives a rich understanding of the common barriers faced, accomplishments and progress achieved in the face of those challenges, and Partners’ relationships to the Network and each other. This paper proposes solutions to common barriers in implementing Network support and coordination activities in year 2 and reflects on the utility of the MRI as a multi-purpose tool for communication and engagement as well as monitoring and evaluating DSHN as a systems-level intervention.

Despite the steady decline in the birth rate among females aged 15–19 in the USA since 1991, rates remain significantly higher compared to other industrialized nations, with disparities across racial and ethnic populations (CDC, 2021; Martin et al., 2019, 2021). Federal funding from the Office of Adolescent Health, the Family and Youth Services Bureau, and the Centers for Disease Control to support growth in the inventory of evidence-based prevention programs has impacted teen pregnancy, but there remains a lack of organized effort to reduce rates in key priority areas where young people continue to experience disparities and have fewer available evidence-based programs (Wilson et al., 2018; Garcia et al., 2022; Office of the Assistant Secretary for Health (OASH), 2020).

Disparities in sexual health outcomes for youth with disabilities are persistent (Baines et al., 2018) and require new and creative approaches to address the many complex and individualized barriers facing this population. Organizations serving the needs of youth with disabilities are more often nonprofit and lack the capacity and expertise to effectively develop and test interventions to move this field forward. Research indicates the need to provide ample time for exploration, development, and systematic evaluation of innovation efforts (Kasper & Clohesy, 2008). While some funding streams are available to rigorously evaluate innovative strategies to reduce teen pregnancy, these initiatives do not typically allow time to explore and develop new ideas (Office of the Assistant Secretary for Health (OASH), 2020).

Little research exists currently on successful methods for building the capacity of service providers to spark innovation in programming. The Teen Pregnancy Prevention Program (TPP) cites Systems Change Theory as the framework for their Innovation and Impact Network Project (IIN; OASH, 2020), an initiative supporting the establishment of Networks to develop new interventions in the area of sexual health education for priority populations. The IIN Project establishes the expectation of Network grantees to “set the conditions” for innovation through leadership, commitment, and a range of intentional and repeatable processes that provide structure and flexibility to support network partners in successfully growing their ideas, whereas innovations without this type of systematic support and collaboration typically fail (Kasper & Clohesy, 2008).

This opportunity requires parallel innovation in thinking and practices for measuring and assessing processes and outcomes that account for complex system-level phenomena (Renger, 2015). System dynamics are at the center of the IIN Project and require an adaptive mixed methods evaluation strategy that draws influence from system and ecosystem mapping and social network analysis but remains focused on learning and improvement (Hargraves, 2010). By regarding networks as systems, IIN grantees can apply these unique practices and approaches to maximize understanding *in context* of the effectiveness of systems-level interventions to meet the need for ground-up approaches to innovative sexual health education programs for priority populations. This paper reviews the findings from data gathered from Partners through a key network tool, the Monthly Reporting Instrument (MRI; See Appendix for instrument), and reflects on its effectiveness in supporting, monitoring, and evaluating progress toward Network goals.

## Project Description

The Disability-inclusive Sexual Health Network (DSHN) Project is a statewide, collaborative project led by SexEdVA at the James Madison University (JMU) Institute for Innovation in Health and Human Services that establishes, coordinates, and supports a network of partners to explore, develop, test, refine, and evaluate innovative interventions that improve optimal health, prevent teen pregnancy, and address sexually transmitted infections (STIs) for youth with disabilities. SexEdVA leads the Network in partnership with the Virginia Department of Education Training and Technical Assistance Center (TTAC), a regional institution supporting services for students with disabilities. The network of Partners that make up the DSHN Project began forming initially during funding proposal development and expanded and solidified at the beginning of the first year of funding through a Call for Innovation Partners (CFIP). Eligible applicants were organizations, teams, and innovators operating in the Commonwealth of Virginia. Eight year 1 partners were selected through the CFIP and finalized as a cohort in March 2021. The year 1 network consisted of small nonprofit community organizations, local affiliates of national nonprofits, and a public institute of higher education.

A key monitoring and communication tool implemented by the network in year 1 is the Monthly Reporting Instrument (MRI), which is completed monthly by Partners one week before their scheduled Monthly Check-in Meeting with DSHN staff to provide a summary of progress on their projects, identify needs for support and coordination, and assess Partners’ relationships to the Network and each other. As this paper will describe, the MRI is a unique tool developed and implemented by DSHN that serves multiple but sometimes conflicting purposes. While the MRI is a deliverable specified in Partner subaward agreements and a key data source for monitoring and evaluating the Network, the primary function of the tool in practice is to support the purpose and goals of the Monthly Check-In Meetings that serve to provide individualized training, technical assistance, and other support to Partners. Data collected using the MRI are utilized to develop Check-In Meeting Agendas, prepare resources, and plan requested training and technical assistance to be delivered in a timely and efficient manner each month to Partners.

At the end of year 1, the DSHN staff and Evaluation Team began a comprehensive review of program data pertaining to questions on the Network Learning Agenda. To enact timely improvements to data collection instruments and make recommendations for adjustments to the Network Management Plan as year 2 partners were being recruited and onboarded, preliminary analysis prioritized the following year 1 Priority Network Learning Questions:

1. What was the overall progress of each Partner during year 1, including summary challenges and achievements?
2. How engaged were Partners with Network activities, processes, and objectives in year 1?
3. How effectively did monitoring and data collection tools identify Partner needs for support and coordination, and capture progress toward Network goals in year 1?

This paper focuses on the preliminary findings from MRI year 1 data and related program records. Our findings provide an understanding of the utility of DSHN’s approach to supporting, monitoring, and evaluating the Network in a systems context. Results are used to adjust the Network Management Plan and assess the usefulness of the MRI as a tool for both communication and engagement, and monitoring and evaluation.

## Method

A mixed methods approach was used to address each of the year 1 Priority Network Learning Questions on the overall progress and engagement of Partners, and to assess the MRI’s utility to inform on progress toward Network Learning Agenda items as the project moves into its second year. The participants were eight sub-awardees contracted by JMU under the Innovation and Impact Network Project Grant as year 1 partners.

### Data Collection and Analysis

The MRI includes 27 closed and open-ended survey items that monitor progress toward project goals at the Partner level, identify areas for training or technical assistance, and assess opportunities and achievements in learning, coordination, and collaboration (see Appendix). A representative from each Partner organization was asked to complete the MRI each month via Google Forms (a data collection website that users can access remotely) in advance of their scheduled Check-In Meeting with DSHN staff. MRI responses, monthly meeting agendas, and staff notes were cataloged in program records to facilitate planning, quality improvement, and follow-up.

Responses to the MRI were exported to a Microsoft Excel database for analysis at the conclusion of 13 months of data collection. Fields containing personal identifiers (name and email) were removed, and partner organization names were replaced with pseudonyms (Partners A through H) before providing the data file to the Evaluation Team for analysis. Partner G submitted more than one MRI in some months due to reporting separately on more than one intervention for this project. Partners A and B combined their projects mid-year and transitioned to one combined MRI submission and monthly meeting. To measure Network engagement, the true count of meetings and MRIs for each Partner was included in the analysis, resulting in a total monthly participation of greater than two (one meeting and one MRI) in some cases. The MRI dataset was analyzed to address year 1 Priority Network Learning Questions through the overall progress of partners, the level of engagement across the network, and the usefulness of the MRI in identifying needs and opportunities to provide support and coordination to Network Partners.

### Quantitative Data Analysis

Excel was used to tabulate frequencies for closed-ended MRI items to examine differences between Partners and trends over time. Correlation analyses were performed on continuous variables to assess the practical significance of relationships between measures of interest. Cases were deleted listwise where one or more of the source variables was missing.

### Qualitative Data Analysis

Qualitative (open text) entries from the MRI dataset were reduced through open coding to identify common themes. Two members of the Evaluation Team independently reviewed each category of open text entries and met to compare coding. Emergent themes were additionally validated through triangulation with data from meeting agendas, notes, and correspondence with DSHN staff. Because Partners frequently addressed common subjects such as collaboration in response to multiple item responses, themes were coded across MRI questions when applicable. Thus, the total number of valid responses for each topic may be greater than the number of submissions. Frequencies of emergent themes were calculated based on the number of descriptive responses, excluding values that were either blank or otherwise indicated as “not applicable.” Excel was utilized to calculate frequency and percentage of coded themes across Partners and MRI submissions.

## Results

A total of 65 MRI submissions were collected across eight year 1 partners between March 9, 2021, and March 16, 2022. On average, submissions were 77% complete (SD = 17.0), with the completion rate of closed-ended items being higher (92.8%, SD = 3.89) compared to open-ended items (61.5%, SD = 19.30). In the absence of skip logic or other instructions within the tool, partners varied in whether they left non-applicable open-ended questions blank or entered text (e.g., “no change” or “N/A”). While participation in the MRI was not 100%, it was consistent, with a moving average of monthly submissions ranging from 5.0 to 5.5 (SD = 0.50) (Table 1).

**Table 1:** Year 1 Overall participation rates, overall and year-end rating of connection, collaboration rates by partner, March 2021–March 2022

	Participation, %			Connection (Score range 1.0–5.0)		Collaboration rate,^*c*^ % (*N* = 13)
Partner	MRI participation (*N* = 13)	Meeting attendance (*N* = 13)	Sum engagement rate (*N* = 26)	Overall connection rating^*a*^* M* (SD)	Year-end connection rating^*b*^	
A	69.2	61.5	57.7	*3.3* (0.45)	3.0	30.8
B	46.2	38.5	57.7	*3.7* (0.90)	4.0	46.2
C	84.6	84.6	84.6	*3.6* (1.36)	5.0	46.2
D	30.8	38.5	42.3	*3.4* (0.58)	2.0	7.7
E	46.2	46.2	65.4	*3.8* (0.00)	4.0	23.1
F	53.8	53.8	69.2	*3.4* (0.53)	4.0	38.5
G	76.9	69.2	80.8	*3.8* (0.79)	5.0	53.8
H	69.2	69.2	84.6	*3.2* (0.71)	4.0	23.1
Average, *M* (SD)	*7.8* (2.38)	*7.5* (2.14)	*17.6* (3.93)	*3.5* (0.23)	*3.9* (0.99)	*32.7* (2.25)
Correlation coefficient with year-end connection rating^*d*^	*r* = 0.713	*r* = 0.640	*r* = 0.686			*r* = 0.822

^a^Two cases were deleted listwise from correlational analysis due to missing data for the connection rating variable for those months

^b^Year-end rating taken from MRI submission closest to the end of the study period for each partner (average month 12, range month 9–13)

^c^Collaboration rate was calculated as the percentage of months in which a Partner reported collaborating with other partners

^d^Pearson’s *r* was calculated for practical significance of the relationship between participation and collaboration metrics and the year-end connection rating

### Overall Progress, Challenges, and Achievements

Partners reported each month on general progress made since the last check-in with an open-ended item and during discussion with DSHN staff during monthly Check-In Meetings. For the scope of this analysis, findings related to overall progress, including common challenges and achievements, were drawn from MRI responses to three items: “Please identify your current progress toward the following [Intervention Road Map] milestones […];” “What accomplishments are you celebrating?” and “What challenges are you experiencing?” Additional qualitative data was coded from responses to the MRI prompt “Please share a brief update of activities and progress over the last month.”

Partners often discussed barriers they were facing to meeting project goals. Partners’ open-ended responses to the MRI item “What challenges are you experiencing?” contained 50 descriptive entries (76.9% response rate). Challenges mentioned ranged from lack of follow-up from potential program sites to delays in filling vacant staff positions. The most common themes mentioned are listed in Table 2 with supporting quotes from Partners and include Limited Time and Staffing (32.0%) and Lack of Community Buy-In (22.0%).

**Table 2:** Themes on the topic of “challenges and accomplishments”

**Challenges themes**	Sample quotes	Frequency, *n* (%)	
		Partners	Responses
1. Time/staffing	“Time is always a challenge in terms of scheduling to meet the project needs…Knowing we are behind w[h]ere we had hoped to be by this point in time.” “Had a Project Coordinator in mind and she is unavailable this summer.” “Delays in publication schedule due to internal staff shortage”	*4* (50.0)	*16* (32.0)
2. Community/partner buy-in	“[A county school system] seemed interested, but they are not returning my emails regarding a meeting” “Lack of response. The fact that Special Ed teachers are overwhelmed and do not want to take on anything additional at this moment.” “Some resistance by [partners] to sexual health trainings as 'not our responsibility’”	*4* (50.0)	*11* (22.0)
3. COVID-19 pandemic	“Most of our [redacted] trainers are unable to do any face-to-face training right now.” “We have not resumed our in person activities because we feel it's too risky for our families.”	*4* (50.0)	*8* (16.0)
4. Technical support needs	“Knowing where to go from here with our survey responses.” “Some problems identifying graphics tool for SPED Manual revisions”	*3* (37.5)	*4* (8.0)
5. Recruiting Participants	“Having [a participant] with IDD comfortable enough to want to attend the sessions. Recruiting has been harder than expected.” “Finding people willing to commit the time is one issue. Finding youth and/or parents/guardians willing to be involved.”	*2* (25.0)	*2* (4.0)

Table contains quotes from responses (*N* = 50) given by partners (*N* = 8) for Select Monthly Reporting Instrument (MRI) Item between March 2021 and March 2022

Fifty-five MRI submissions (84.6% response rate) included an open-ended response to the question “What accomplishments are you celebrating?” Accomplishments celebrated by Partners ranged from general progress on project objectives to more specific accomplishments or milestones, such as filling a vacant staff position (Table 3): planning and development milestones (38.2%) and people and partnerships (38.2%).

**Table 3:** Themes on the topic of “accomplishments”

**Accomplishment themes**	Sample quotes	Frequency, *n* (%)	
		Partners	Responses
1. Development and planning milestones	“Selection of a curriculum, collaboration, and the support we receive from DSHN staff!” “We are ready to open registration for our training and have all become trained.” “Closing our survey and getting useful feedback!”	*7* (87.5)	*21* (38.2)
2. People and partnerships	“The consistency of the participants coming to our workshops is something to celebrate! Each week, we had youth keep coming back!” “We love our youth advisory panel and celebrate them!” “The fact that schools and the Department of Ed want to partner!”	*5* (62.5)	*21* (38.2)
3. Pilot testing	“Having completed the pilot sessions!” “We did one training! and got good feedback.” “…We are thrilled with how [our workshop] went. We had 7 consistent participants and feel as if our workshops were insightful for these…men.”	*5* (62.5)	*16* (29.1)
4. Material creation	“Love the parent guide we have created!” “We were able to bring a film crew in from MN and complete the filming for the first 5 lessons.”	*5* (62.5)	*10* (18.2)
5. Network opportunities/connections	“Collaboration and support we receive from the DSHN staff!”	*3* (37.5)	*8* (14.5)

Table contains quotes from responses (*N* = 55) given by partners (*N* = 8) for select Monthly Reporting Instrument (MRI) item between March 2021 and March 2022

### Partner Engagement

Engagement, which is often measured by examining behavioral (e.g., dedication, participation) and cognitive (e.g., positive affect, feelings of connection) components (e.g., Reschly & Christenson, 2012; Shuck et al., 2012; Wefald et al., 2011), was assessed using three metrics: participation, connection, and collaboration. Measures used to assess these dimensions for each Partner were 1) monthly meeting attendance and MRI response rate; 2) response to the MRI item “How connected do you feel to the Network as a whole?” and, 3) response to the MRI item “In what ways did you collaborate with other Network Partners this month?”.

### Participation

Participation was measured through attendance of Monthly Check-In Meetings and submission of MRIs. Meeting participation was measured by the number of meetings each Partner participated in compared to the thirteen scheduled during the study period. Partners attended on average 7.5 meetings (SD = 2.17, range: 5–11) in year 1. The median number of meetings convened each month across all partners was four (*M* = 4.6, SD = 2.13, range: 1–8). Year 1 partners submitted an average of 8.5 (*N* = 13, 65.4%, SD = 2.83) MRIs during the study period, with participation ranging from four submissions to 13 for Partner G’s multi-project submissions. The median number of MRI submissions received each month was five (*M* = 5.2, SD = 1.25, range: 3–7).

A sum rate of participation was calculated by combining meeting attendance with MRI participation (out of a total of 26 scheduled communications in year 1 through 13 monthly meetings and 13 MRIs). On average, Partners had a participation rate of 73% (score of 18.9; range 11–25; see Table 1 for component and sum participation rates by month and partner). The lowest point of participation for both Check-In Meetings and MRI submissions (sum of five) was in June 2021, during which Partners were invited to opt out of monthly meetings and reporting in lieu of attending the Network Annual Summit that month. Excluding June from the analysis, the average sum participation rate of each Partner was 76% (*N* = 24, SD = 3.96).

### Connection

**Fig. 1:**
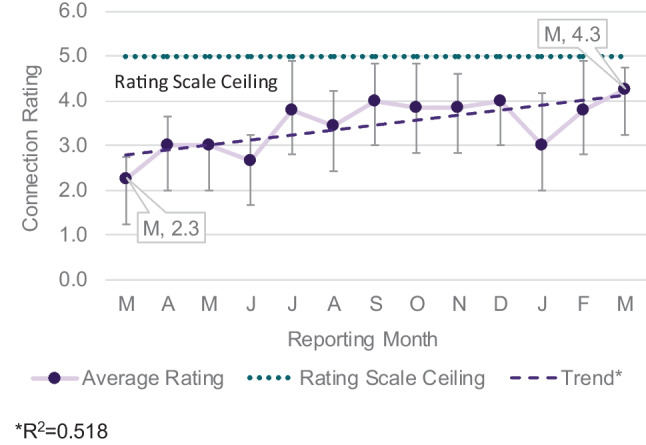
Average Monthly Connection Rating and Change Across Year 1 for All Innovation Partners, March 2021-March 2022

Partners submitting an MRI were asked to rate their feeling of connection to the Network on a scale of 1 to 5, with response options ranging from *not very* (1) to *very* (5) connected. Overall, Partners rated their feeling of connection across year 1 on average as a 3.4 (range 2.5–4.0, *SD* = 0.55). Monthly connection rating across all partners for the 13-month study period showed an overall moderate trend of increased connection from the start to end of year 1, with the average rating increasing from 2.3 in month 1 to 4.3 in month 13 (*R*^2^ = 0.563, SD = 0.61; Fig. 1).

### Collaboration

To measure internal collaboration, responses to the MRI item “In what ways have you collaborated with other Network partners this month?” were coded as “Yes (reported collaboration)” and “No (did not report collaboration).” The average number of Partners who reported collaboration each month was 2.6 (SD = 1.15, range: 1–4; Table 1). Each Partner reported collaborating on average 4.4 months out of the 13 reporting months (SD = 1.22, range: 3–6). The rate of collaboration for each partner was calculated as a percentage of the months they reported collaborating. The average rate of collaboration across all partners for the reporting period was 33.7% (SD = 9.37, range: 23.1–46.2). Data from this MRI item were also coded for the number of instances of collaborative activities (emails, phone calls, meetings, or other activities) mentioned by each Partner. For example, a response mentioning an email communication with one Partner and a phone call with another was coded as two instances. Partners reported on average 4.4 instances of collaboration (SD = 1.87 range: 1–7) during the reporting period. During an average month, partners across the Network reported 6.6 collaborative activities (SD = 3.90, range: 1–14).

### Engagement

To examine the relationship between participation, collaboration, and feelings of connection to the network as aspects of engagement, correlational analyses were conducted across each measure. Connection was positively correlated independently with both total year 1 meeting attendance (Pearson’s *r* = 0.640) and MRI submissions (*r* = 0.713) with a moderate effect size (Table 1). Individual and sum measures of engagement did not have significant correlation with the year 1 average connectivity rating (*r* values of − 0.013 and − 0.115, respectively). The correlation between overall sum engagement and year-end connection rating resulted in a Pearson’s *r* of 0.686, indicating a positive relationship with a moderate practical effect size and suggesting that partners who had higher participation throughout the reporting year also reported higher year-end feelings of connection. Collaboration was similarly correlated with MRI and meeting participation, with the positive correlation between collaboration and sum participation having a moderate effect size (*r* = 0.549) indicating higher participation was related to more frequent collaboration across partners.

**Fig. 2:**
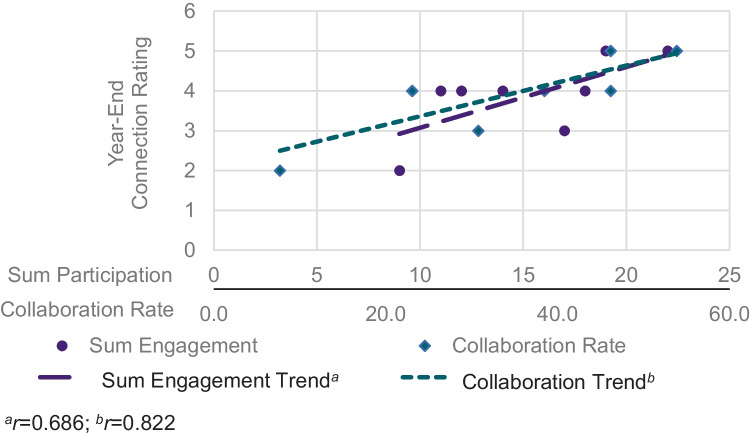
Correlation of Sum Participation and Collaboration Rate withYear-EndConnection Rating by Partner

Finally, we found that connection and collaboration were the most strongly correlated with each other with a large effect size (*r* = 0.822), indicating that partners who reported higher year-end feelings of connection also more frequently reported collaborating with others. These relationships between participation (Sum Engagement), connection (year-end connection rating) and collaboration (collaboration rate) are depicted in Fig. 2.

### Collaboration and Networking

To better understand what collaboration looked like for year 1 partners, two additional MRI items were coded for emerging themes on the topics of internal collaboration and external networking activities. Partners are encouraged not only to collaborate and network with each other, but to share their work with stakeholders outside the network to support the growth and strength of the Network. Responses to the questions “In what ways have you collaborated with other network partners this month?” and “Have you shared any information about your project outside the Network?” were analyzed for a summary of common activities Partners engaged in as part of their work with the network.

Fifty-one MRIs (78.5%) contained a response indicating partners had collaborated in some way with other network partners in the past month. Partners offered examples such as sharing contacts and joint efforts such as bringing staff from two partner organizations together to attend a shared training (Table 4): sharing expertise (62.1%) and project collaboration (17.2%).

**Table 4:** Themes on the topic of “external and internal networking”

**External networking**	Sample quotes	Frequency, *n* (%)	
		Partners	Responses
1. Not Yet	“When the project is completed we will.” “Not yet. We are still in the development stage.”	*7* (87.5)	*21* (41.2)
2. Yes	“The [redacted] is featuring the project in our upcoming newsletter, plan to add to our website and Facebook page.” “Actually yes! [Redacted] and I presented at the Association for Positive Behavior Support virtual conference…and were asked about how we intended to expand [our project]…”	*5* (62.5)	*17* (33.3)
**Internal networking**	Sample quotes	Frequency, *n* (%)	
		Partners	Responses
1. Sharing expertise	“Met with [partner] for ideas as she has done a lot of virtual workshops.” “Coordinated with [partner] on suggested legislative language.”	*6* (75.0)	*18* (62.1)
2. Project collaboration	“We are working with a new partner to pilot our intervention” “I'm working with [partner] to do some training on LGBTQ + terminology and talking about porn with young people.”	*4* (50.0)	*5* (17.2)
3. Sharing contacts or resources	“Contacted [partner] for referral to local graphic arts resources for SPED manual.” “Our Pre-consumer survey has been shared on Facebook by [partner]” “We have been sharing the upcoming workshops [partner] is hosting with our networks”	*3* (37.5)	*4* (13.8)

Quotes and frequency of external networking themes mentions by partner (*N* = 8) and responses (*N* = 51) for Select Monthly Reporting Instrument (MRI) Item, March 2021–March 2022; internal networking themes by partner (*N* = 8) and submission (*N* = 29)

On the topic of external networking, 57 (87.7%) MRI submissions contained a response to the item on sharing information outside of the Network. However, only 28% of submissions contained a description of actual networking activities. Five Partners mentioned external networking they had engaged in that month a total of 17 times (33.3%; Table 4). External networking activities Partners described included social media posts, newsletter articles, marketing, and conference presentations. Notably, 88% of Partners (*n* = 7) stated in at least one MRI submission they had not shared anything about their project externally that past month or do not plan to until their project is further along and/or complete.

### Needs for Support and Coordination

Multiple items from the MRI were reviewed for responses indicating a need or request from Partners for any type of training, technical assistance, support, or coordination that could be provided or facilitated by DSHN staff. Two main items were analyzed for each general topic of needs or requests around Support (“What question(s) do you need answered in your partner check-in?” and “What training, technical assistance, or support would you find most useful at this stage?”) and Coordination (“Do you have any ideas to increase connections among the Network or ways in which we can utilize our meeting spaces together?” and “Is your organization offering any opportunities or seeking anything right now that we can share with our broader audience?”), with additional themes coded from more general open-ended items such as “Additional Information” where entries found there pertained to support and coordination needs.

To increase connection and make better use of network resources and relationships partners frequently identified a need for more opportunities for small group discussion with other partners (40.9%), particularly those who had commonalities across program designs or target populations. Partners offered suggestions such as forming topical discussion groups and adding informal asynchronous options for online engagement. Of note, 18% of responses related to this topic mentioned satisfaction with current trainings and resources (Table 4): small group zoom meetings (27.3%); and happy with existing opportunities (18.2%).

Partners were also asked to indicate the areas in which they felt they could use more support from DSHN staff or other Partners. Although the response rate on this topic was low (47.7% across two main MRI items), three common themes arose indicating that Partners most often needed assistance with program evaluation, development of materials, and networking and collaborating with other Partners (Table 5): evaluation and data collection (60.0%); material development (50.0%); and networking for collaboration (50.0%).

**Table 5:** Themes on the topic of “needs and requests for coordination and support”

**Coordination themes**	Sample quotes	Frequency, *n* (%)	
		Partners	Responses
1. Zoom meetings with smaller groups	“Collaborate with like projects in breakout sessions” “Grouping project types in breakout sessions i.e., all youth interventions meet”	*3* (37.5)	*6* (27.3)
2. Happy with existing opportunities	“The quarterly meetings are most helpful” “I'm looking forward to our network meeting so that I can hear [from other Partners].”	*3* (37.5)	*4* (18.2)
3. Online networking space	“Some sort of group page that we could all share resources on…so all partners could communicate in a less formal way. Partners could even upload their materials to share…”	*2* (25.0)	*2* (9.1)
**Support themes**	Sample quotes	Frequency (%)	
		Partners	Responses
1. Material development	“Curriculum development and script writing” “Assistance in Sex Ed topic gaps, script development and evaluation methodologies” “Input from school staff who will make decisions about curriculum”	*5* (62.5)	*5* (50.0)
2. Evaluation and data collection	“Feedback on evaluations when they are complete.” “Assistance framing Focus Group questions for 2 populations: teens, young adults.” “Review of our survey questions once a draft is completed”	*4* (50.0)	*6* (60.0)
3. Networking/coordination	“Networking with other partners” “Networking to find an audience for another pilot group of youth with IDD.”	*3* (37.5)	*5* (50.0)

Quotes and frequency of mentions of coordination themes by partner (*N* = 22) and responses (*N* = 31) for Select Monthly Reporting Instrument (MRI) Item, March 2021–March 2022; support themes by partner (*N* = 8) and responses (*N* = 10)

## Discussion

The MRI provided a wealth of information about the experience of partners during the first year through 351 descriptive text responses, and an additional 656 responses to closed-ended questions. This input from partners, while primarily gathered for the purpose of providing support and guidance to the network, is valuable for summarizing progress of the project over time and for assessing the impact of Network-level activities on Partners’ success. Findings from this study will inform planning and improvements for year 2 to fuel progress in the face of reported challenges, adjust monitoring and evaluation activities, and ultimately increase the strength and complexity of DSHN as a connected and collaborative system.

### Partner Progress in Year 1

Unsurprisingly, the community-based organizations that make up the DSHN mentioned availability of staff and time as the most frequent barrier to making progress toward project goals and objectives. However, partners experienced unanticipated challenges in securing buy-in and commitment from stakeholders for both program sites and potential participants. Although in some cases commitment from stakeholders was difficult due to resource constraints, partners reported several instances of resistance to invest in their project, citing sexual health education was “not [their] responsibility.” One partner said about challenges around recruiting participants: “[h]aving [a participant] with IDD comfortable enough to want to attend the sessions [has been a challenge]. Recruiting has been harder than expected.”

### Partner Engagement in the Network

Participation in monthly reporting and check-in meetings varied widely by Partner (*M* = 70.2%, range 42.3–96.2). If a Partner needed to cancel a meeting, they were encouraged to still complete the MRI for that month. Despite this, there was lower overall participation in the MRIs than the monthly meetings (62.5% compared to 77.9%). Program records show that in some months, a meeting and/or MRI were replaced with an email or deferred altogether at the Partner’s request. Partner D had the lowest participation rate (34.6%) and has a stated preference for email communication over formal reports and meetings. However, this Partner provides frequent updates to staff and is furthest in developing their intervention, having completed several pilot sessions by the end of year 1. These observations indicate that some measures of progress and engagement may not be captured accurately by the MRI due to lower overall participation in the reporting tool. Email and phone communication between partners and staff were not considered in this analysis, nor were network-wide opportunities for engagement and connection such as quarterly network meetings and the annual summit. A more global review of those metrics may provide better insight into the degree to which partners are interacting with the network and their preferred methods and platforms.

Partners reported strong agreement with feeling connected to the network by the end of year 1, with six of the eight partners describing their feeling of connection as “strong” or “very strong.” The overall positive trend of the connection rating over the year indicates progress toward fostering engagement and collaboration among partners. However, a ceiling effect on this measure may limit the continued utility of this metric and the ability to detect the impact of network-level interventions on partners who already report a high connection rating.

The connections that partners celebrated were diverse and complex, spanning multiple coded themes in the analysis. Partners described numerous achievements around project teamwork, engagement with stakeholders and participants, and connection and collaboration with network partners and staff. During the study period, two partners chose to combine their efforts, noting that they were excited about “[t]he collaboration and combination of our projects into one and the opportunity to work with an inspiring colleague!” The need for ongoing support for Partners to continue making connections with each other was apparent in our findings, with assistance networking with other Partners and community stakeholders described five times by three separate Partners as a technical support need.

In contrast, the apparent lack of reported sharing on their project outside of the network raises the question of whether Partners are in fact not talking about their projects and need skill development on ways to share, network, and celebrate aspects of a project that are very much under development; or whether they are sharing and just need clarification on how to identify these activities as “networking.” Many partners may find themselves unaccustomed to engaging with their stakeholders on an idea under development, but such communication is essential to growing support for partner interventions that can contribute to sustaining the goals of the network after grant funding ends.

While these findings provide insight on partner engagement, it should be acknowledged that the role of the MRI for contractual progress reporting presents limitations in its utility to accurately capture the authentically experience of partners, and in particular, their subjective feedback on network efforts. Specifically, the collection of data on collaboration and connection for this analysis required the presence of a datapoint on participation in the MRI so it is unknown how partners who did not participate in the MRI are feeling or interacting with other partners. The validity and reliability of the MRI as a measure of a complex construct such as engagement needs further exploration.

### Approaches for Monitoring and Evaluation

The low participation in the MRI is an indication that Partners may find this tool burdensome or ineffective for communicating progress and support needs. This, in addition to the questions raised above, suggests the MRI may benefit from revisioning or repurposing to ensure quality data for evaluating progress and impact across the project. The MRI is an incomplete “record” of the progress partners have achieved over the first year. For every instance where an MRI was skipped or replaced by an email, a summary of progress including challenges and achievements is missing from the data and not accounted for in the findings of this study. Some redundancy in the tool also became apparent during analysis, in cases where key themes on progress, challenges, engagement, and collaboration were coded across multiple responses.

## Conclusion

Partner-identified needs and requests for support and coordination were often immediately actionable by the DSHN staff, such as requests for guidance and technical assistance. In many cases, the DSHN staff were alerted to these needs by the MRI and could prepare assistance to be implemented during the subsequent check-in meeting. This observation is key to the argument for maintaining some sort of monthly monitoring tool that can inform the agenda for meetings to make the best use of that time. Based on the findings of this study, SexEdVA will implement several improvements to both communication, and monitoring and evaluation activities in year 2 of the project. These preliminary adjustments focus on key aspects of the network learning agenda and evaluation goals. Namely, training and support for partners to impact progress on their projects, activities to facilitate connection and collaboration within and outside of DSHN, and effective monitoring and data collection strategies to continue tracking progress toward network goals and learning questions.

Training and resources in year 2 will respond to common reported challenges, including training on talking to parents and educators as stakeholders and potential community partners. Resources on branding and marketing will encompass how to share project information outside the network and build branding for partners’ interventions to facilitate this important communication.

Changes to Network communication and monitoring approaches will address the burdensomeness of the MRI, and the utility and validity of some item categories. A streamlined version of the MRI will focus on monthly identification of support needs to be addressed at check-in meetings. Bigger-picture topics such as engagement, collaboration, and progress toward Learning Agenda objectives will be moved to an anonymous Quarterly Partner Survey. Additional opportunities for communication between partners will be created through a dedicated Slack channel (a web-based messaging app), break-out rooms during quarterly network meetings, and periodic “cluster meetings” that bring together a small group of partners in lieu of individual check-in meetings.

The Quarterly Partner Survey will include improved measures for engagement and separate these important metrics from required reporting activities. Connection will be better assessed using a multi-item scale based on existing validated scales to address the observed ceiling effect of the current measure. Additional data sources for participation and collaboration that account for all the ways Partners are interacting with staff and each other outside of monthly meetings and reports (email and phone communications, impromptu meetings, etc.), will be incorporated into future analyses to give a more accurate picture of participation and engagement.

Beyond the scope of the project, lessons learned from year 1 of this initiative contribute to developing insights on successfully building and managing network-based partnerships. Using a network approach to support capacity building and innovation across diverse organizations has the potential to accelerate innovative solutions to complex issues such as teen pregnancy among underserved populations, including youth with disabilities. The ongoing development and application of effective systems-based strategies and accompanying tools for supporting, monitoring, and evaluating these efforts is essential to moving forward teen pregnancy prevention efforts in general and in developing programs and interventions influenced by systems thinking.

## Appendix

Unless otherwise indicated, the survey response option is an open-ended text box allowing up to 32,000 characters.

### Disability-inclusive Sexual Health Network (DSHN) Monthly Reporting Instrument (MRI)

1. Email Address.
2. Today’s Date.
3. Partner Name.
4. Name of Person Completing this Report.
5. Please share a brief update of activities and progress over the last month.
6. What challenges are you experiencing?
7. What accomplishments are you celebrating?
8. Is your organization offering any opportunities or seeking anything right now that we can share with our broader audience? For example: perhaps you’re conducting a focus group and need participants to give feedback, or maybe you’re offering a workshop and want to publicize it. Please briefly describe and we will follow up with you.
9. What question(s) do you need answered in your partner check-in?
10. Have you shared any information about your project outside the Network (meetings with other organizations, newsletters, blog posts, conferences, etc.? If so, please let us know who/where. We would love to know!
11. Please identify your current progress towards the following milestones of intervention exploration and development (Phase 1 of Intervention Roadmap). Select one: Not yet started, In progress, Complete, N/A
   Milestones: Ideation/Prototyping, Created plan for involving diverse co-creators, Established Theory of Change, Intervention Materials Developed, Materials Submitted for Review, Intervention Plan Developed, IRB Plan Developed
12. If you would like to provide additional information/clarification about progress towards Phase 1 milestones, please do so here (optional):
13. Please select the impact area most closely aligned with your intervention. Select one: Youth, Parents / Caregivers, Teachers / Educators
14. Can your intervention help to answer any of the following learning questions? Select all that apply: [List of Learning Questions by Impact Area]
15. Please briefly describe any new progress being made towards answering any of the learning questions you selected.
16. What resources have you utilized in the exploration and development of your intervention? Select all that apply: [List of Network-related resources, updated as new resources become available], Other (specify)
17. What training, technical assistance, or support would you find most useful at this stage?
18. How connected do you feel to the Network as a whole? Select one: 1 – Not at all connected, 2, 3, 4, 5 – Very connected
19. In what ways have you collaborated with other Network partners this month? (email, meetings, resource sharing, etc.)
20. Do you have any ideas to increase connections among the Network or ways in which we can utilize our meeting spaces together?
21. Additional Information: (open text response)
